# Predictive value of CD73 expression for the efficacy of immune checkpoint inhibitors in NSCLC

**DOI:** 10.1111/1759-7714.13346

**Published:** 2020-02-15

**Authors:** Hidenobu Ishii, Koichi Azuma, Akihiko Kawahara, Takashi Kinoshita, Norikazu Matsuo, Yoshiko Naito, Takaaki Tokito, Kazuhiko Yamada, Jun Akiba, Tomoaki Hoshino

**Affiliations:** ^1^ Division of Respirology, Neurology, and Rheumatology, Department of Internal Medicine Kurume University School of Medicine Kurume Japan; ^2^ Department of Diagnostic Pathology Kurume University Hospital Kurume Japan

**Keywords:** CD73, epidermal growth factor receptor, immune checkpoint inhibitor

## Abstract

**Background:**

CD73 induces the dephosphorylation of adenosine monophosphate converting it to adenosine, enabling malignancies to escape from immune surveillance. Although CD73 overexpression has been reported to be a poor prognostic factor in several malignancies including non‐small cell lung cancer (NSCLC), its predictive relevance in NSCLC patients receiving immune checkpoint inhibitors is unknown. The present research was conducted to investigate the prognostic significance of CD73 expression in NSCLC patients receiving immune checkpoint inhibitors (ICIs).

**Methods:**

We screened 91 patients with advanced or recurrent NSCLC who received immune checkpoint inhibitors. CD73 expression was evaluated immunohistochemically using tissue specimens obtained just before treatment with ICIs.

**Results:**

Analysis of progression‐free survival (PFS) and overall survival (OS) in relation to several levels of CD73 expression (1%, 10%, 30%, and 50%) showed that both tended to be more favorable as expression of CD73 increased. PFS and OS were longer for patients in whom at least 50% of the tumor cells expressed CD73 than for those in whom <50% of the tumor cells did so. In patients who were positive for *EGFR* mutation, immune checkpoint inhibitors were significantly more effective in those with high CD73 expression, whereas CD73 expression did not significantly affect the efficacy in patients with *EGFR* mutation‐negative NSCLC. Furthermore, CD73 expression was predictive factor for the PFS independent of PD‐L1 expression in patients with *EGFR* mutation.

**Conclusions:**

High CD73 expression may predict a favorable response to ICIs in NSCLC patients, especially those harboring *EGFR* mutations.

**Key points:**

Significant findings of the study: In patients who were positive for EGFR mutation, immune checkpoint inhibitors (ICIs) were significantly more effective in those with high CD73 expression, whereas CD73 expression did not significantly affect the efficacy in patients with EGFR mutation‐negative NSCLC.

What this study adds: High CD73 expression may predict a favorable response to immune checkpoint inhibitors in NSCLC patients, especially those harboring EGFR mutations.

## Introduction

Non‐small cell lung cancer (NSCLC) is the leading cause of cancer death worldwide.^1^ Immune checkpoint inhibitors (ICIs) have been recently used as a new strategy for treatment in various malignancies.^2^, ^3^ Several clinical trials demonstrated that treatment with antiprogrammed cell death 1 (PD‐1)/PD‐ligand 1 (PD‐L1) antibodies showed a promising efficacy.^4^, ^5^, ^6^, ^7^

Recently, it was suggested that overexpression of CD73 creates an immunosuppressive microenvironment in NSCLC tumors. CD73, known as 5′‐nucleotidase or ecto‐5′‐nucleotidase, catalyzes the dephosphorylation of extracellular adenosine monophosphate (AMP) to adenosine and inorganic phosphate. The released extracellular adenosine inhibits the antitumor function of T cells and induces T cell apoptosis, thus helping tumors to escape immune surveillance.^8^, ^9^ CD73 is frequently expressed in a large number of malignancies and is correlated with poor patient survival.^10^, ^11^, ^12^, ^13^ Streicher *et al*. have reported that epidermal growth factor receptor (*EGFR*) mutation‐positive cell lines show significantly high CD73 expression, and that expression of CD73 is decreased by EGFR inhibition in NSCLC cell lines.^14^ However, the prognostic value of CD73 expression in NSCLC patients, including those with *EGFR* mutation‐positive NSCLC, receiving ICIs is unclear. This retrospective study aimed to evaluate the prognostic significance of CD73 expression in NSCLC patients receiving ICIs.

## Methods

### Patients

We retrospectively examined consecutive NSCLC patients who had been treated with immune checkpoint inhibitors (ICIs) at Kurume University Hospital between February 2016 and September 2018. Of these, 91 patients who had adequate biopsy tissues with enough tumor cells were enrolled into the present study. Among these patients, we screened 25 with advanced or recurrent *EGFR* mutation‐positive NSCLC and 66 with *EGFR* mutation‐negative NSCLC who had received ICIs. All *EGFR* mutation‐positive patients were treated with ICIs after developing resistance to EGFR‐TKI therapy.

We followed the provisions of the Declaration of Helsinki and obtained study approval from the Institutional Review Board of Kurume University Hospital.

### Tumor samples

Formalin‐fixed paraffin‐embedded tumor slices obtained by biopsy before treatment with immune checkpoint inhibitors (ICIs) were used for analysis. *EGFR* mutations were detected using the Cobas EGFR Mutation Test (Roche Diagnostics Deutschland GmbH, Mannheim, Germany).

### Immunohistochemical analysis for CD73 and PD‐L1 expression

The sections were mounted onto slides and incubated with antirabbit monoclonal antibodies against CD73 and PD‐L1 (Cell Signaling Technology, Danvers, MA, USA) for immunohistochemical (IHC) analysis using a BenchMark XT slide staining system (Ventana Automated Systems, Inc., Tucson, AZ, USA). For both PD‐L1 and CD73 IHC analysis, each specimen had to contain more than 100 viable malignant cells and the percentage of stained malignant cells in the entire area of the tumor (tumor proportion score; TPS) was determined.

### Statistical analysis

The association between CD73 expression level and the efficacy of immune checkpoint inhibitors (ICIs) was evaluated regarding the overall response rate (ORR), progression‐free survival (PFS), and overall survival (OS). ORR was defined as the proportion of patients who achieved a complete response or partial response according to the Response Evaluation Criteria in Solid Tumors (ver. 1.1). PFS was estimated as the period from the start of treatment with ICIs to the date of disease progression or death due to any cause. OS was defined as from the start of treatment until the death or the date of last follow‐up. Survival curves were analyzed by the Kaplan‐Meier method and a log‐rank test was performed to analyze the significance of differences between two groups. All statistical analyses were conducted using JMP version 12 software (SAS Institute Inc., Cary, NC, USA).

## Results

### Patient characteristics

The characteristics of the enrolled patients are shown in Table 1. We observed positive expression of PD‐L1 in 56 of the patients (67.0%). Additionally, 25 patients (27.5%) had *EGFR* mutations. All patients positive for *EGFR* mutation received immune checkpoint inhibitors (ICIs) subsequently developed resistance to EGFR‐TKI treatment. A total of 55 patients received nivolumab, 33 patients received pembrolizumab, and three patients received atezolizumab as an ICI.

**Table 1:** Patient characteristics

Characteristics	Number of patients	%
Age		
Median	68	
Range	41–89	
Sex		
Male	66	72.5
Female	25	27.5
Smoking		
Never	21	23.1
Former/current	70	76.9
Histology		
Nonsquamous	68	74.7
Squamous	23	25.3
Performance status		
0–1	74	81.3
2‐	17	18.7
PD‐L1 expression		
Negative	30	33.0
Positive	61	67.0
*EGFR* mutation		
Mutated	25	27.5
Wild	66	72.5
Immune‐checkpoint inhibitor (ICI)		
Nivolumab	55	60.4
Pembrolizumab	33	36.3
Atezolizumab	3	3.3

EGFR, epidermal growth factor receptor; PD‐L1, programmed cell death ligand‐1.

### Survival analysis according to CD73 expression level

**Figure 1:**
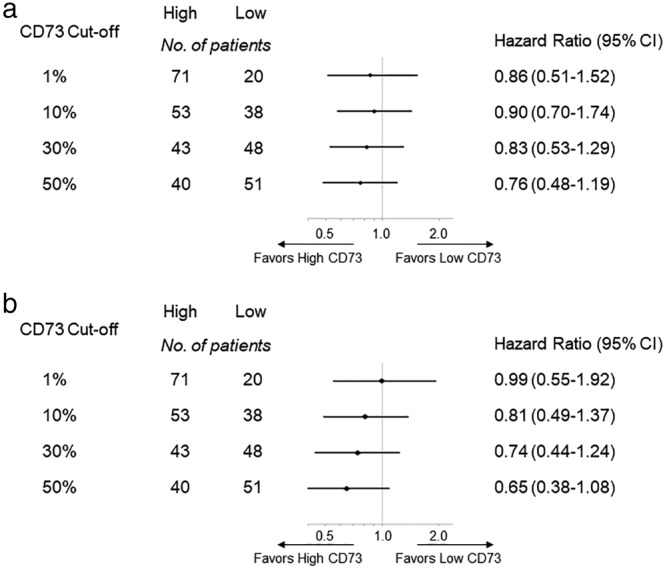
Forest plot for (**a**) PFS and (**b**) OS in NSCLC patients receiving immune checkpoint inhibitors in relation to several cutoff levels of CD73.

**Figure 2:**
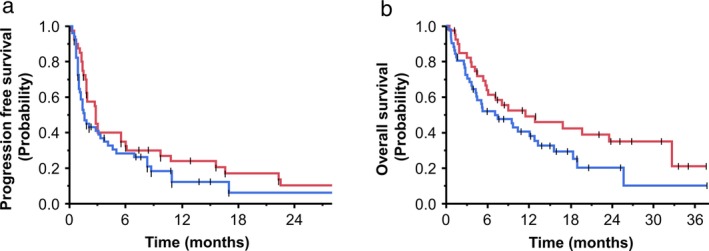
Kaplan‐Meier curves for (**a**) PFS and (**b**) OS after treatment of NSCLC patients with immune checkpoint inhibitors in relation to CD73 expression.

For several cutoff levels of CD73 expression, we divided the patients into two groups and compared the PFS and OS between them for each level. Both PFS (Fig 1a) and OS (Fig 1b) tended to become more favorable as the expression of CD73 increased. Patients with a TPS of ≥50% for CD73 expression had longer PFS and OS than those with a CD73 TPS of <50% (Fig 2a: median PFS in the high and low CD73 groups, 2.8 months and 1.6 months, respectively; *P* = 0.225. Figure 2b: corresponding median OS 11.5 months and 7.1 months, respectively; *P* = 0.099).

### Efficacy of immune checkpoint inhibitors in *EGFR* mutation‐positive patients

**Figure 3:**
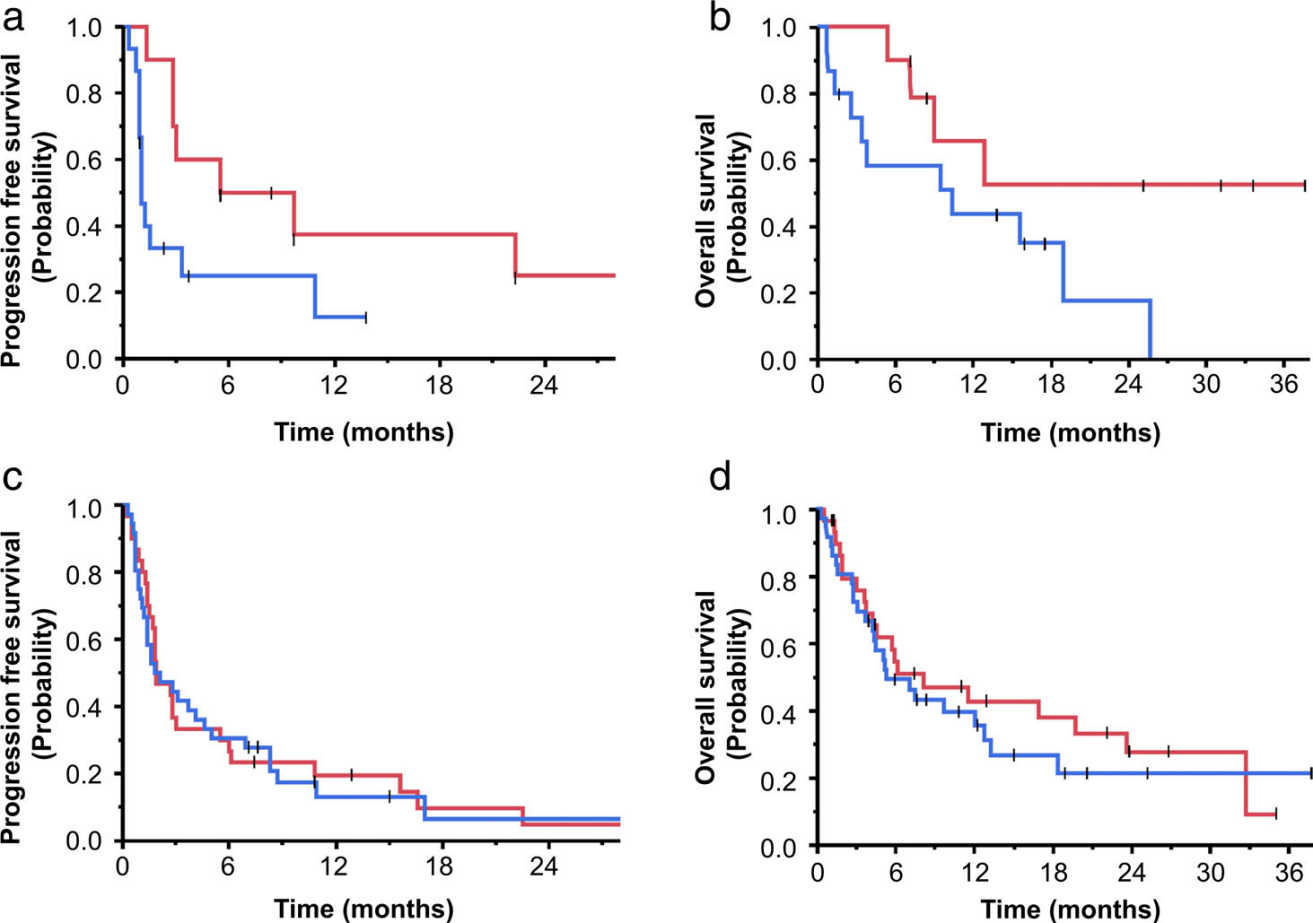
Kaplan‐Meier curves for PFS and OS after treatment with immune checkpoint inhibitors in patients with EGFR mutation‐positive (PFS; **a**, OS; **b**) and ‐negative (PFS; **c**, OS; **d**) NSCLC with high and low CD73 expression.

Among patients positive for *EGFR* mutation, the ORR in those receiving immune checkpoint inhibitors (ICIs) was significantly higher in patients with high CD73 expression than in those with low CD73 expression (60.0% vs. 6.7%, respectively, *P* = 0.007; Table 2). PFS was significantly longer in patients with high CD73 expression than in those with low expression after treatment with ICIs (median PFS, 7.6 vs. 1.0 months, respectively, *P* = 0.043; Fig 3a), and the OS also tended to be longer in those with high CD73 expression (median OS not reached vs. 10.4 months, respectively, *P* = 0.077; Fig 3b).

**Table 2:** Tumor response to immune‐checkpoint inhibitors (ICIs)

	Tumor response				
	PR	SD	PD	Response rate	*P*‐value
All patients					
High CD73	13	7	20	32.5%	0.042
Low CD73	7	15	29	13.7%	
*EGFR*‐mutated					
High CD73	6	0	4	60.0%	0.007
Low CD73	1	5	9	6.7%	
*EGFR*‐wild					
High CD73	7	7	16	23.3%	0.547
Low CD73	6	10	20	16.7%	

EGFR, epidermal growth factor receptor; PD, progressive disease; PR, partial response; SD, stable disease.

### Efficacy of immune checkpoint inhibitors in *EGFR* mutation‐negative patients

In patients negative for *EGFR* mutation, expression of CD73 was not associated with the ORR for immune checkpoint inhibitor (ICI) treatment (23.3% in patients with high CD73 expression vs. 16.7% in those with low CD73 expression, *P* = 0.547; Table 2). Furthermore, the level of CD73 expression had no significant effect on PFS and OS for *EGFR* mutation‐negative patients (median PFS 1.9 months in patients with high CD73 expression vs. 2.0 months in those with low expression, *P* = 0.997; Fig 3c, median OS 8.1 months in patients with high expression vs. 5.3 months in patients with low expression, *P* = 0.510; Fig 3d), unlike the situation in patients with *EGFR* mutation.

### Association between CD73 expression and patient characteristics

The association between patient characteristics and CD73 expression are shown in Table 3. High CD73 expression and PD‐L1 expression were significantly correlated in all patients. In *EGFR* mutation‐positive patients, high expression of CD73 was also associated with PD‐L1 expression (Table SS1). CD73 expression was not correlated with age, gender, smoking status, or performance status.

**Table 3:** Association between CD73 and patient characteristics

		CD73 expression		
Characteristics	Number of patients	High	Low	*p*‐value
Age				
< 70	47	21	26	0.886
> 71	44	19	25	
Sex				
Male	66	29	37	0.996
Female	25	11	14	
Smoking				
Never	21	10	11	0.700
Former/current	70	30	40	
Performance status				
0–1	74	33	41	0.798
2‐	17	7	10	
PD‐L1 expression				
Negative	30	7	23	0.007
Positive	61	33	28	
*EGFR* mutation				
Mutated	25	10	15	0.640
Wild	66	30	36	

EGFR: epidermal growth factor receptor; PD‐L1, programmed cell death‐ligand 1.

## Discussion

As immune checkpoint inhibitors (ICIs) have been recently used as a new strategy for treatment in NSCLC, it is necessary to identify biomarkers that are able to predict the efficacy to anti‐PD‐1/PD‐L1 treatment. Although the results of preliminary phase III clinical trials of anti‐PD‐1/PD‐L1 treatment for NSCLC have suggested that expression of PD‐L1 can predict the response to this type of treatment, expression of PD‐L1 is not considered to be a perfect biomarker. In this study, high expression of CD73 in NSCLC patients receiving ICIs tended to be more prognostically favorable (CD73 TPS ≥50%) than low expression (TPS <50%), particularly in patients with *EGFR* mutation‐positive NSCLC.

CD73 has been reported to be an important predictor of poor prognosis in several cancers.^10^, ^11^, ^12^, ^13^ To investigate the prognostic relevance of CD73 expression in *EGFR* mutation‐positive NSCLC patients untreated with ICIs, we further screened 67 patients with stage I‐III *EGFR* mutation‐positive NSCLC who had undergone complete resection. Patients with high CD73 expression had a relatively shorter disease‐free survival and OS than those with low CD73 expression (Figure SS1). As far as we are aware, no correlation between CD73 expression and efficacy of treatment with ICIs has been previously investigated for individuals with NSCLC. The present study demonstrated that high expression of CD73 was a factor predictive of a positive response to ICIs in *EGFR* mutation‐positive NSCLC patients. Furthermore, in patients with *EGFR* mutation‐negative NSCLC, expression of CD73 was not associated with the outcome of treatment with ICIs, unlike the situation in patients who were *EGFR* mutation‐positive. Our findings appear to be consistent with those of previous preclinical studies, which have shown that the mechanism of CD73‐induced immune tolerance involves the EGFR signaling pathway.^15^

The present study demonstrated that high CD73 expression was significantly correlated with PD‐L1 expression in also patients with *EGFR* mutations. The results of preliminary phase III clinical trials of anti‐PD‐1/PD‐L1 therapy against NSCLC have suggested that PD‐L1 expression is predictive of the response to this type of treatment.^4^, ^6^ However, it has been reported that PD‐L1 expression is an imperfect biomarker for predicting the efficacy of ICIs in *EGFR* mutation‐positive NSCLC.^16^ We analyzed the predictive value of PD‐L1 expression in enrolled *EGFR* mutation‐positive patients. PD‐L1 expression was not associated with the PFS of ICIs (median PFS was 1.5 months in the PD‐L1‐negative group and 3.0 months in the PD‐L1‐positive group, *P* = 0.714) and OS (median OS was 15.6 months in the PD‐L1‐negative group and 10.4 months in the PD‐L1‐positive group, *P* = 0.592) as previously reported.^16^ Furthermore, multivariate analysis showed that CD73 expression was a predictive factor for the PFS independent of PD‐L1 expression (Table 4).

**Table 4:** Multivariate analysis for PFS and OS in patients with *EGFR* mutation

	PFS			OS		
	HR	95% CI	*P*‐value	HR	95% CI	*P*‐value
CD73	0.245	0.070–0.823	0.023	0.285	0.066–1.082	0.065
PD‐L1	0.504	0.158–1.613	0.243	0.674	0.204–2.307	0.519

CI, confidence interval; HR, hazard ratio; OS, overall survival; PD‐L1, programmed cell death‐ligand 1; PFS, progression‐free survival.

Recently, a new therapeutic product designed to target CD73 has been developed. MEDI9447, a human monoclonal antibody, selectively binds to CD73 and inhibits its ectonucleotidase activity.^17^ On the basis of the addictive activity of MED9447 with ICIs revealed in preclinical studies, a phase I trial is currently ongoing to investigate the safety and efficacy of MEDI9447 as monotherapy, or in combination with durvalumab for advanced solid tumors. In addition, a clinical trial of MEDI9447 plus osimertinib is currently being planned for patients with previously treated *EGFR* mutation‐positive NSCLC. The results obtained from those studies might help to clarify the clinical significance of CD73 in immune target treatment for patients with *EGFR* mutation‐positive NSCLC.

This study had some limitations. A major point was that the number of patients analyzed was relatively small. Next, the data was collected retrospectively. Further prospective clinical researches are therefore warranted to clarify the value of CD73 expression, and the therapeutic effect of anti‐PD‐1/PD‐L1 treatment.

In conclusion, we have showed that high CD73 expression is correlated with favorable clinical efficacy of ICIs in patients with *EGFR* mutation‐positive NSCLC who have developed resistance to EGFR‐TKI treatment. These findings may have important implications for immunotherapy in such patients.

## Disclosure

The authors have no potential conflicts that are relevant to this manuscript.

## Supporting information


**Figure S1.** Kaplan‐Meier curves for disease‐free survival (DFS) and overall survival (OS) in patients after complete resection of stage I–III *EGFR* mutation‐positive NSCLC according to CD73 (DFS; **a** and OS; **b**).

**Table S1.** Association between CD73 and patient characteristics in *EFGR* mutation‐positive patients
