# Supplementary figures and images for: Predictive value of CD73 expression for the efficacy of immune checkpoint inhibitors in NSCLC
Source: Thorac Cancer. 2020 Feb 15;11(4):950–5. doi: 10.1111/1759-7714.13346 (PMC7113063; doi:10.1111/1759-7714.13346)

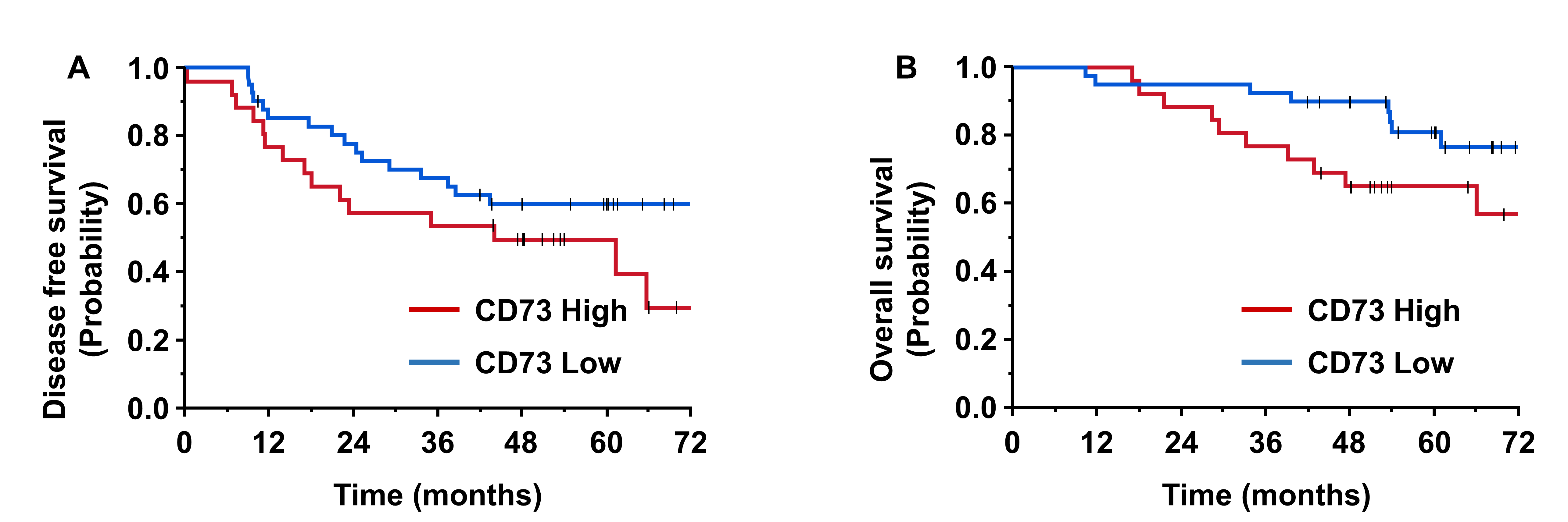

Supplement: Supplementary file 1 — Figure S1. Kaplan‐Meier curves for disease‐free survival (DFS) and overall survival (OS) in patients after complete resection of stage I–III EGFR mutation‐positive NSCLC according to CD73 (DFS; a and OS; b). [file TCA-11-950-s001.tif]
