# Supplementary material for: Predictive value of CD73 expression for the efficacy of immune checkpoint inhibitors in NSCLC
Source: Thorac Cancer. 2020 Feb 15;11(4):950–5. doi: 10.1111/1759-7714.13346 (PMC7113063; doi:10.1111/1759-7714.13346)
Supplement: Supplementary file 2 — Table S1. Association between CD73 and patient characteristics in EFGR mutation‐positive patients [file TCA-11-950-s002.docx]

**Supplementary Table 1. Association between CD73 and patient characteristics in *EFGR* mutation-positive patients**

| Charateristics | Number of patients | CD73 expression | | *p*-value |
| --- | --- | --- | --- | --- |
|  |  | High | Low |  |
| Age |  |  |  |  |
| < 70 | 12 | 5 | 7 | 1.000 |
| > 71 | 13 | 5 | 8 |  |
| Sex |  |  |  |  |
| Male | 10 | 5 | 5 | 0.442 |
| Female | 15 | 5 | 10 |  |
| Smoking |  |  |  |  |
| Never | 13 | 4 | 9 | 0.428 |
| Former/Current | 12 | 6 | 6 |  |
| Performance status |  |  |  |  |
| 0-1 | 22 | 10 | 12 | 0.250 |
| 2- | 3 | 0 | 3 |  |
| PD-L1 expression |  |  |  |  |
| Negative | 9 | 1 | 8 | 0.041 |
| Positive | 16 | 9 | 7 |  |

PD-L1: programmed cell death-ligand 1.
